# Investigating the Therapeutic Potential of *Tamarix aphylla* Leaf Extract Against Toxicity Caused by Graphene Nanosheets in *Cirrhinus mrigala*

**DOI:** 10.1002/vms3.70918

**Published:** 2026-04-10

**Authors:** Muhammad Asad, Kashif Ali, Tehseen Fatima, Nagina Rehman, Abeer Kazmi, Md. Fakhrul Islam, Aneela Nijabat, Asma Ashraf, Ihsan Ullah, Ayesha Siddiqa, Juan Pedro Luna‐Arias, Gabriela Medina‐Pérez, Amir Ali

**Affiliations:** ^1^ Department of Zoology Division of Science & Technology University of Education Lahore Lahore Pakistan; ^2^ Department of Zoology University of Mianwali Mianwali Pakistan; ^3^ The State Key Laboratory of Freshwater Ecology and Biotechnology Institute of Hydrobiology Chinese Academy of Sciences Wuhan Hubei PR China; ^4^ University of Chinese Academy of Sciences Beijing PR China; ^5^ Department of Fisheries Jamalpur Science and Technology University Jamalpur Bangladesh; ^6^ Department of Botany University of Mianwali Mianwali Pakistan; ^7^ Department of Zoology Wildlife & Fisheries Pir Mehr Ali Shah Arid Agriculture University Rawalpindi Pakistan; ^8^ Department of Botany PMAS Arid Agriculture University Rawalpindi Pakistan; ^9^ Department of Cell Biology and Nanoscience and Nanotechnology PhD Program Center for Research and Advanced Studies of the National Polytechnic Institute Mexico City Mexico; ^10^ Institute of Agricultural Sciences Autonomous University of the State of Hidalgo Hidalgo Mexico

**Keywords:** *Cirrhinus mrigala*, graphene nanosheets toxicity, *Tamarix aphylla* leaf, therapeutic potential

## Abstract

**Background and Objective:**

Graphene nanosheets (GNS) have found more applications in biomedical and industrial uses, but exposure to GNS can cause adverse health outcomes, such as oxidative stress, inflammatory responses, and tissue injury, depending on their dose and physicochemical characteristics. This study evaluates the potential of *Tamarix aphylla* leaf extract to mitigate GNS toxicity in *Cirrhinus mrigala*.

**Methods:**

Conducted under controlled conditions, the research comprised two phases. Phase I determined the 96‐h LC_50_ of GNS as 1213.72 mg/L, with subsequent exposures set at 10%, 20%, and 30% of LC_50_ (121.37, 60.58, and 40.45 mg/L). Phase II involved a 14‐day co‐treatment of fish exposed to 60.58 mg/L GNS with 25, 50, and 75 mg/L of the extract.

**Results:**

High‐performance liquid chromatography (HPLC) identified key bioactive compounds in the extract, including ferulic, chlorogenic, gallic, p‐coumaric, and caffeic acids, along with malic, citric, and oxalic acids. Malic acid was the predominant compound. Histopathological analysis revealed GNS‐induced damage in the liver, kidney, and intestine, such as necrosis, sinusoidal dilation, and glomerulopathy. However, these effects were significantly reduced in extract‐treated groups, highlighting the protective role of antioxidants and organic acids in the extract.

**Conclusion:**

The findings suggest that *T. aphylla* leaf extract effectively reduces GNS toxicity, offering a promising therapeutic strategy for mitigating the environmental impact of graphene‐related pollutants.

## Graphical abstract

*Tamarix aphylla* leaf extract significantly alleviated graphene nanosheet–induced toxicity in *Cirrhinus mrigala*, reducing oxidative tissue damage and restoring organ integrity. Rich in antioxidant compounds, the extract offers a promising natural strategy for mitigating the biological impacts of graphene‐related pollutants.

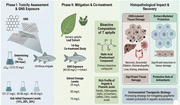

## 1 Introduction

Graphene is a two‐dimensional nanomaterial composed of a single layer of carbon atoms arranged in a hexagonal lattice, known for its exceptional mechanical strength, electrical conductivity, large surface area, and unique physicochemical properties (Xu et al. 2013). Graphene captures significant attention in the scientific world due to its diverse applications. In scientific and industrial domains, graphene nanoforms provide promising materials due to their unique structure and properties (Mortazavi et al. 2021). The applications of graphene nanosheets (GNS) are diverse and impactful, spanning areas such as electronics, optoelectronics, energy storage and conversion, composite materials, and biomedical applications (Lee et al. 2008; Bae et al. 2010; Schwierz 2010; Wang et al. 2010; Yang et al. 2010; Yang et al. 2012). GNS can enter biological systems through oral, inhalation, and dermal exposure routes, where they have been reported to induce cell membrane disruption, mitochondrial dysfunction, and histopathological alterations (Sanchez et al. 2012; Ou et al. 2016; Jin et al. 2025). These toxic responses are strongly influenced by the physicochemical properties of GNS, which are governed by synthesis routes such as oxidation, reduction, and chemical exfoliation, thereby modulating their biological interactions and toxicity profiles (Tonelli et al. 2015). These nanosheets vary varying terms of toxicity depending on their shape, size, surface chemistry, and biological system under study (Ganguly et al. 2018). As their applications continue to grow, a significant impact has been observed on the environment and human health. Nanotoxicology focuses on the effects of nanomaterials on living organisms (Sanchez et al. 2012). Their expansion and diversity have become crucial for assessing their potential toxic effects to ensure environmental safety. The possible way for the entry of nanoparticles into aquatic ecosystems is through direct discharge, accidental spill, or runoff from land‐based sources (Krug and Wick 2011). The nanomaterials have been transformed by different factors such as pH, temperature, and interaction with other organisms (Ding et al. 2022). Across different trophic levels, graphene affects living organisms and raises concerns about the potential consequences of GNS (Zhao et al. 20210. Although graphene‐based materials are often considered biocompatible, they can still cause adverse effects in mammalian systems (Tonelli et al. 2015).

GNS are broadly utilised nanomaterials that have special characteristics, but can cause toxicity because of the formation of reactive oxygen species (ROS), resulting in oxidative stress, mitochondrial dysfunction, and tissue damage (Jarosz et al. 2016; Ou et al. 2016; Jin et al. 2025). Phytochemicals present in *T. aphylla*, including phenolics and flavonoids, are capable of scavenging free radicals and reducing ROS accumulation, thereby mitigating oxidative damage induced by graphene exposure. Moreover, these bioactive compounds may suppress inflammatory signalling by inhibiting ROS‐mediated activation of inflammatory pathways, leading to reduced cellular and tissue inflammation (Sanchez et al. 2012). Medicinal plants are used to minimise the effects of toxicity due to their therapeutic benefits. *T. aphylla* also stands out among medicinal plants, holding pharmacological significance (Narayanan et al. 2020). This plant has been extensively studied for its chemical composition and pharmacological properties. The important bioactive compounds in this plant are phenolic acids, flavonoids, and tannins, which have diverse biological activities, including anti‐inflammatory (Bahramsoltani et al. 2020), antimicrobial (Orabi et al. 2022), anti‐diabetic (Mahfoudhi et al. 2016), Antihyperglycemic (Ullah et al. 2017), wound healing (Gul et al. 2022), anti‐cytotoxic (Alshehri et al. 2021), antioxidant (Suleiman 2019), and analgesic (Qadir et al. 2014).

This plant cures many ailments, such as cough (Alshehri et al., 2021), asthma (Qadir et al. 2014), wound (Gul et al. 2022), jaundice (Marwat et al. 2009), hair loss (Qasem 2015), abdominal pain (Altemani et al. 2024), rheumatism (Iqbal et al. 2017), and measles (Fakchich et al. 2024). These benefits indicate potential therapeutic applications of this plant that further correlate with reducing or minimising toxicity induced by various environmental contaminants. *Cirrhinus mrigala*, a freshwater fish native to the Indo‐Pak subcontinent, occupies a crucial place in aquatic ecosystems and the food chain (Ghayyur et al. 2021). Its economic significance in the commercial fishing industry is noteworthy, emphasising the importance of studying its vulnerability to environmental stressors and toxic substances (Kundu et al. 2026). This study aims to investigate the protective role of *T. aphylla* leaf extract against toxicity caused by GNS.

## 2 Materials and Methods

### 2.1 Graphene Nanosheets and Suspension Preparation

The production of GNS for inducing toxicity in fish took place at the Physics Laboratory, University of Education, Lahore (Jauharabad Campus), Punjab, Pakistan. To create the aqueous suspension of nanosheets, a mixture was sonicated in 5 mL of distilled water for 2 h using a sonicator (model: S‐DS‐6, Stalwart) to ensure the uniform dispersion of nanosheets. The methodology employed was based on the procedure outlined by Wazir and Kundi (2016). The synthesised GNS underwent characterisation using Raman spectroscopy and field emission scanning electron microscopy (FESEM). This allowed for a detailed analysis of the properties and structure of the nanosheets.

### 2.2 Preparation of *Tamarix aphylla* Leaf Extract

The aerial parts, specifically leaf, of *T. aphylla* were collected from moderately mature trees along Muzaffargarh Road in Jauharabad, District Khushab, during February and March of 2023. Following collection, the leaves underwent a thorough cleaning, after which they were left to dry in the shade. Once dried, the leaves were transformed into a powder using either herb grinders. The powdered plant material was immersed in methanol for one month. The resulting extracts were then subjected to filtration using Whatman filter paper No. 101 with a pore size of 11 microns and a diameter of 12.5 cm (Jan et al. 2019).

### 2.3 Physico‐Chemical Parameters

Throughout this acclimatisation (7 days) phase, the fish were nourished with a commercial fish food product (Oryza Organics Private Ltd. of Hong Kong) containing 30% crude protein, 6% crude fat, and 5% crude fibre. Any deceased fish or those displaying unusual symptoms were promptly excluded from the experimental setup to maintain the integrity of the study. Feeding occurred twice a day at 4% of their body weight. A daily regimen involved changing approximately 80% of the water in the semi‐static test tanks. The dissolved oxygen levels were maintained within the range of 6.5–7.4 mg/L, while the pH levels were kept within the range of 6.7–7.2. Special attention was given to maintaining water temperature, which was carefully controlled at a constant 25°C throughout both the acclimatisation and experimental phases. Parameters such as total hardness, total dissolved solids, and ammonia concentration underwent regular monitoring to ensure they remained within optimal levels for the well‐being of the fish.

### 2.4 Analysis of Phytochemicals by HPLC

The chromatographic column facilitated the separation of compounds within the 4 g extract. The separation process was executed using an HPLC system equipped with a UV‐Vis detector, and an autosampler was employed to inject a small volume (10–20 µL) of the *T. aphylla* extract. The identification of phytochemicals within the extract was accomplished based on their retention periods, and their concentrations were determined by comparing peak areas to standard calibration curves.

### 2.5 Model Organism

The fish *C. mrigala*, commonly referred to as Mori, was procured with the support of the Department of Fisheries, Government of Punjab, Chashma, Mianwali. A total of 70 individuals were transported for LC_50_ experiments, and 25 individuals were transported for pharmacological experiments. The fish were desensitised using clove oil following the method of Taylor and Roberts 1999. In a 1‐L beaker filled with tap water, 2 to 3 drops of clove oil were added to sedate the fish before they were dissected by research students who had received proper training following the protocol of Gupta and Mullins 2010.

### 2.6 Experimental Phases

The study comprises two distinct phases. In the initial phase, the lethal concentration (LC_50_) of GNS was determined, while the second phase focused on assessing the ameliorative potential of *T. aphylla*.

### 2.7 Phase I: Experimental Grouping

The fish population (1st experiment) was randomly divided into seven groups, each exposed to varying concentrations of GNS in water, namely 250, 500, 750, 1000, 1250, and 1500 mg/L (Table 1). Fish were transported to small glass aquaria for LC_50_ determination after acclimatisation (2 weeks). GNS were utilised at doses of 0, 121.37, 60.58, and 40.45 mg/L. The exposure time was 96 h, and the temperature, dissolved oxygen level, and pH were all the same as during the acclimatisation time. To determine the LC_50_ value using probit analysis, the dead fish were noted in each concentration. A total of 3 doses of GNS were obtained through the LC_50_ value: High dose (121.37), Medium dose (60.58), and Low dose (40.45) (Table 2).

**TABLE 1:** Phase I: Experimental grouping (exposure of *C. mrigala* to varying doses of GNS).

Groups	Dose GNS mg/L	Mortality	Total
A	0	0	10
B	250	0	10
C	500	1	10
D	750	2	10
E	1000	3	10
F	1250	5	10
G	1500	7	10

**TABLE 2:** Fractioned doses of GNS calculated from LC_50_.

Fractions	Calculations	Experimental doses (mg/L)
10th	1213.72/10	121.37
20th	1213.72/20	60.58
30th	1213.72/30	40.45

### 2.8 Phase II: Experimental Grouping

This experiment was conducted to investigate the ameliorative effects of *T. aphylla* methanolic extract. A total of 5 *C. mrigala* were used as experimental animals, with an average weight of 5 ± 2 g. Fractions corresponding to the 10th, 20th, and 30th parts of the LC_50_ were obtained, with a medium fraction employed both independently and in combination with different doses (25, 50, and 75 mg/L) of *T. aphylla*. This co‐treatment occurred over 14 days and involved five groups, each comprising five individuals (*n* = 5 per group). The fish were randomly divided into four groups according to LC_50_ (Lethal Concentration, 50%) fractional values. Group C served as control, while Groups G, G1, G2, and G3 were administered 0 + 0, 60.58 + 0, 60.58 + 25, 60.58 + 50, 60.58 + 75 mg/L of GNS and *T. aphylla*, respectively, for 4 days (Table 3).

**TABLE 3:** Phase II: Experimental grouping‐control group, group contains only GNS, and three groups contain the same dose of GNS with low, medium, and high doses of TAE.

Groups	GNS + *T. aphylla* (mg/L)
C	0 + 0 mg/L
G	60.58 mg/L
G1	60.58 + 25 mg/L
G2	60.58 + 50 mg/L
G3	60.58 + 75 mg/L

### 2.9 Histology

Preserving selected organs involved immersing them in a 10% formalin solution. Initially, the organs were subjected to a 1‐h tap water cleaning in the washing step. Subsequently, a 3‐h dehydration process ensued, involving various methanol concentrations (70%, 80%, 90%, and 100%). To ensure optimal tissue clarity, a clearing step was followed, employing a 1:1 mixture of methanol and xylene for 30 min, followed by an additional 30 min in 100% xylene. Infiltration and embedding were accomplished by preparing blocks for microtomy, with tissues infiltrated and embedded in molten paraffin. Cutting was performed to obtain 5‐µm‐thick sections using a microtome. Staining with haematoxylin and eosin enhanced visibility and contrast in the sections. Computer‐assisted microscopy facilitated the scoring and detailed examination of histological features and organ alterations in the prepared slides.

### 2.10 SEM and Raman

The morphology of the nanoparticles was investigated using a field emission scanning electron microscope (FE‐SEM; Hitachi S‐4800, Japan). The samples were placed on conductive carbon tape, on mica sheets, or directly on the aluminium sample holder. Images were taken at an accelerated voltage between 1 and 3 kV and a current of 10 µA from COMSATS University Lahore.

Raman analysis of GNS was performed at Deakin University, Australia, using a Raman microscope (Renishaw InVia‐001). The instrument was equipped with a 1064 nm fibre‐optic laser with a maximum laser power of 50 mW and an adjustable power control ranging from 0% to 100%. Raman spectroscopy was employed to obtain the characteristic vibrational fingerprint of GNS, enabling the evaluation of structural order, defect density, and crystallinity.

### 2.11 Statistical Analysis

The results of phytochemicals of *T. aphylla* were analysed by one‐way analysis of variance (ANOVA) using SPSS version 25. *p* Value < 0.05 was considered statistically significant.

## 3 Results

### 3.1 Characterisation of Nanoparticles

**FIGURE 1:**
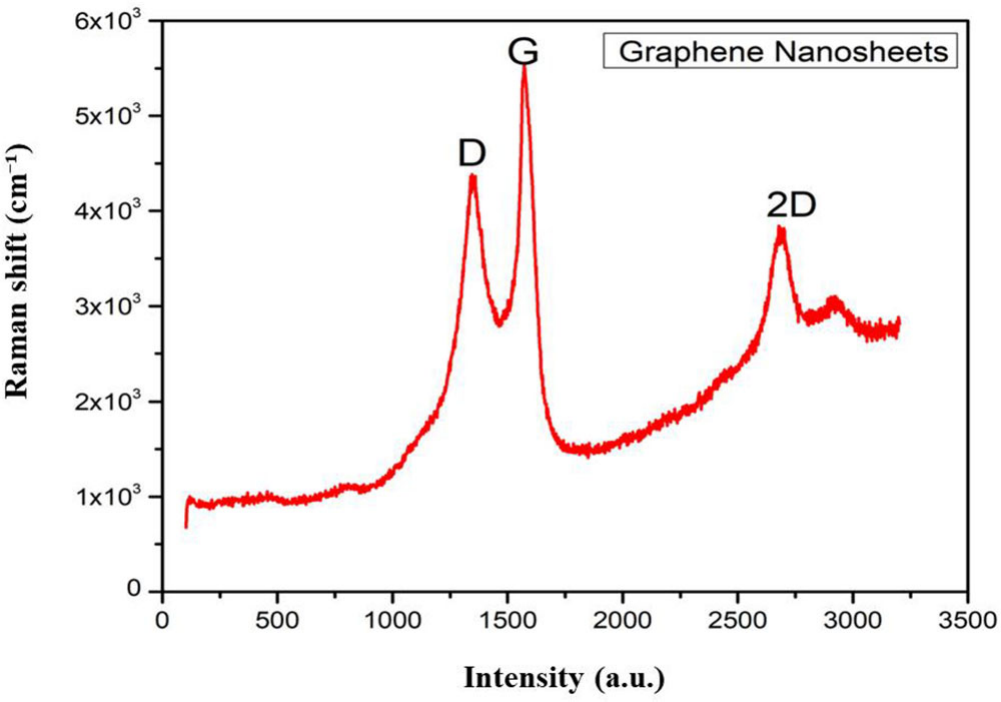
Graph represents Raman spectroscopy of graphene nanosheets showing D, G, and 2D Bands.

The characterisation of nanoparticles is effectively illustrated through Raman spectra, where intensity is depicted on the *y*‐axis and wavelength on the *x*‐axis (Figure 1). The presence of a ‘D peak’ in the Raman spectrum serves as an indication of structural faults or instability within graphene. A larger D peak suggests an elevated level of instability. Conversely, a distinct and well‐defined G peak functions as an indicator of a well‐ordered, crystalline carbon lattice, providing insights into the quality and arrangement of carbon‐based materials. The 2D peak offers information on the overall number of carbon layers present in the material, distinguishing between a single layer (monolayer), a few layers (few‐layer graphene), or multiple layers.

### 3.2 Scanning Electron Microscopy

**FIGURE 2:**
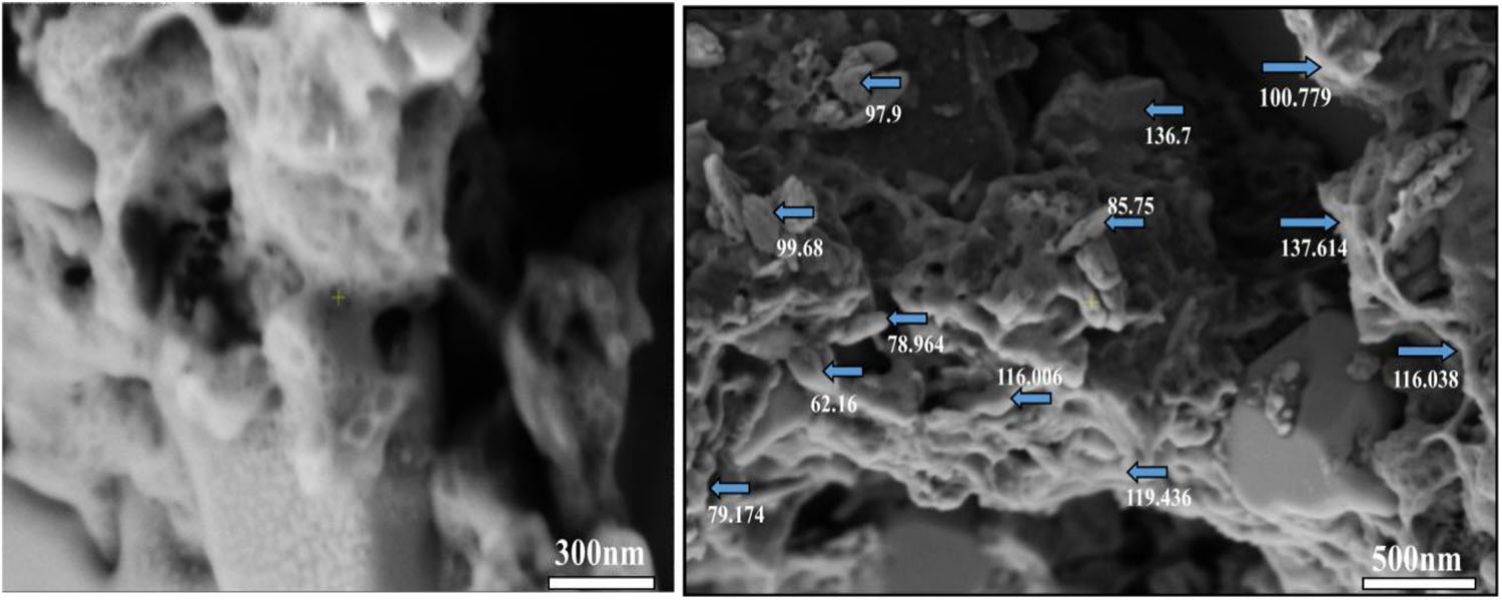
Scanning electron microscopy (SEM) images showing the surface morphology and particle size distribution of the synthesized nanoparticles. The nanoparticles exhibit irregular, agglomerated structures with measured particle sizes ranging from approximately 62.2 nm to 137.6 nm, and an average particle size of about 102.5 nm. Arrows indicate representative nanoparticle size measurements. Scale bars represent 300 nm (left panel) and 500 nm (right panel).

Scanning electron microscopy (SEM) was employed to further characterise GNS, revealing a diverse range of lengths, spanning from 62.2 to 137.6 nm (Figure 2). The mean length of GNS was determined to be approximately 102.5 nm, indicating variability within the nanosheet population. The examination also unveiled the presence of wrinkles and folds, contributing to the complex surface morphology observed in the GNS. These detailed characterisations provide valuable insights into the structural and morphological features of the nanoparticles under study.

### 3.3 Phytochemical Screening

Table 4 outlines the quantities of various compounds measured in parts per million (ppm), providing a comprehensive view of their composition. Malic acid takes the lead with the highest quantity at 58022.93 ± 0.41 ppm, while sinapic acid registers the lowest concentration at 2.735 ± 0.006 ppm. Notable compounds in the intermediate range include ferulic acid, gallic acid, vanillic acid, and quercetin, with respective quantities. Caffeic acid and chlorogenic acid are measured slightly lower, followed by cinnamic acid and P‐coumaric acid. In the acid category, oxalic acid leads at 6560.13 ± 0.64 ppm, followed by citric acid, methylmalonic acid, and malonic acid. Succinic acid is present at 629.12 ± 0.64 ppm, and fumaric acid has the lowest concentration among the acids at 98.065 ± 0.006 ppm. Kaempferol stands out with a quantity of 535.06 ± 0.004 ppm, underscoring its significance in the overall composition.

**TABLE 4:** HPLC results showing phytochemicals (phenolic acids, organic acids, and kaempferol) present in *T. aphylla* methanolic extract.

Phytochemicals	Compounds	Quantity (PPM)
Phenolic acids	Ferulic acid	15.275 ± 0.00645
	Gallic acid	11.122 ± 0.00645
	Vanillic acid	11.0525 ± 0.00479
	Quercetin	10.915 ± 0.00645
	Caffeic acid	8.15 ± 0.06455
	Chlorogenic acid	7.775 ± 0.00645
	Cinnamic acid	7.475 ± 0.00645
	P‐coumaric acid	4.715 ± 0.00645
	Sinapic acid	2.735 ± 0.00645
Organic acids	Malic acid	58022.93 ± 0.40825
	Oxalic acid	6560.13 ± 0.6455
	Citric acid	1247.46 ± 0.6455
	Methylmalonic acid	1176.7 ± 0.6455
	Malonic acid	1028.39 ± 0.6455
	Succinic acid	629.12 ± 0.6455
	Fumaric acid	98.065 ± 0.00645
Kaempferol	Kaempferol	535.065 ± 0.00408

### 3.4 LC_50_ Values

**FIGURE 3:**
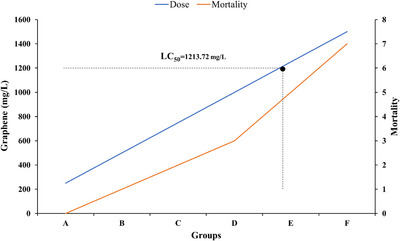
The graph represents a probit analysis to estimate the LC_50_ concentration of graphene nanosheets.

The LC_50_ (median lethal concentration) value for GNS was determined to be 1213.72 mg/L. In the control group (G0), where the concentration of GNS was 0 mg/L, no fatalities were observed (Figure 3). However, as the concentration of GNS increased from G1 to G6, a corresponding rise in the number of deaths occurred, highlighting a dose‐dependent response.

### 3.5 Histopathology Observations

**FIGURE 4:**
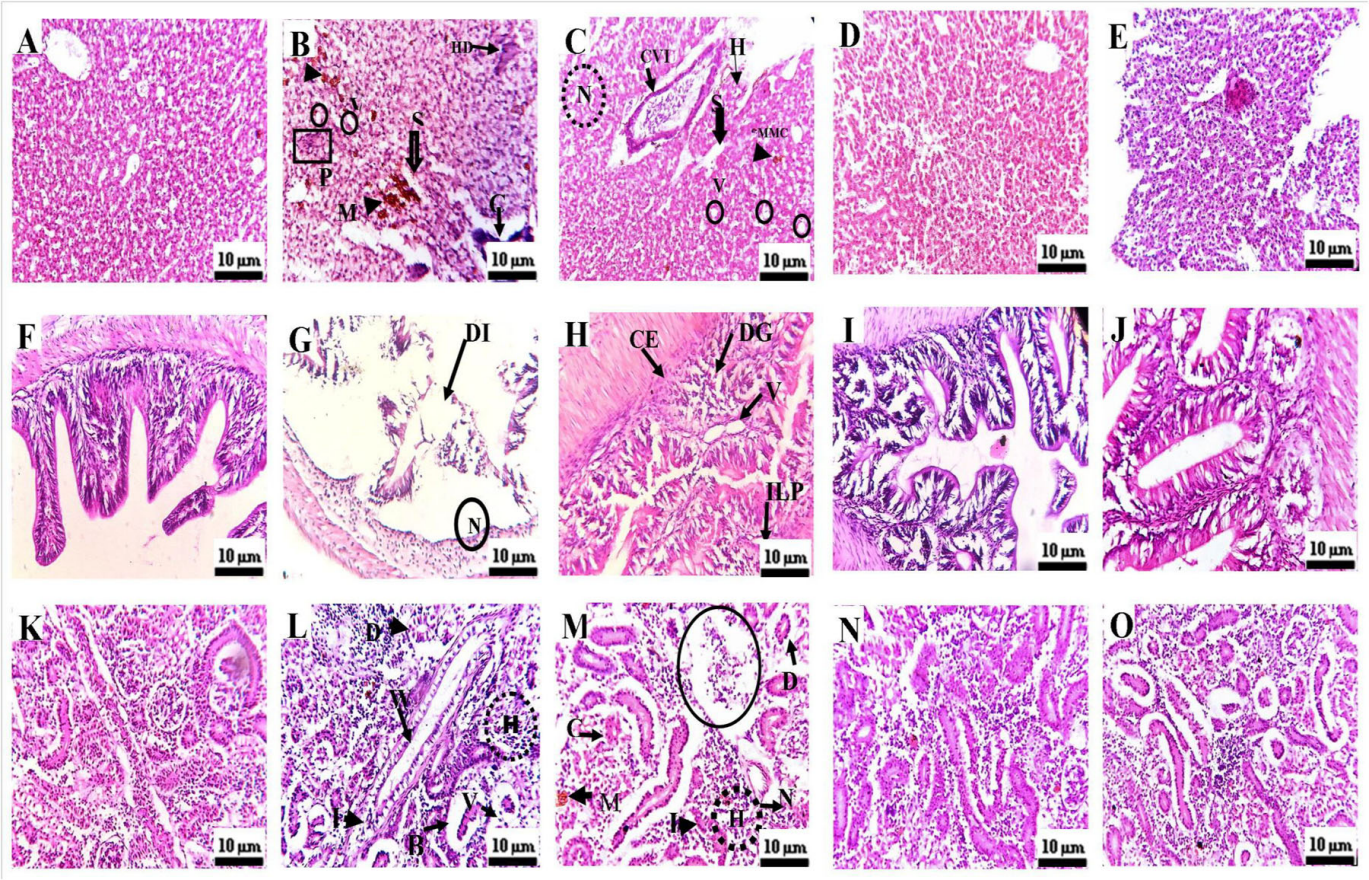
(A–E) Liver histopathology: (A) Control group (C) showing normal hepatic architecture; (B, C) Groups G (GNS 60.58 mg/L) and G1 (*T. aphylla* 25 mg/L combined with GNS 60.58 mg/L; no recovery) exhibiting marked histopathological alterations, including necrosis (N), central vein injury (CVI), hydropic degeneration (HD), sinusoidal dilation (SD), melanomacrophage centres (MMC), vacuolation (V), and pyknotic nuclei (PN); (D, E) Groups G2 and G3 treated with *T. aphylla* leaf extract (50 and 75 mg/L, respectively) in combination with GNS 60.58 mg/L, showing substantial hepatic recovery and restoration of normal tissue architecture. (F–J) Intestine histopathology: (F) Control group (C) showing normal Intestinal architecture; (G, H) Groups G (GNS 60.58 mg/L) and G1 (*T. aphylla* 25 mg/L combined with GNS 60.58 mg/L; no recovery) exhibiting marked histopathological alterations, including necrosis (N), collapsed epithelial layer (CE), degenerated goblet cells (DG), intact lamina propria (IL), vacoulation (V), destroyed intestinal integrity (DI); (I, J) Groups G2 and G3 treated with *T. aphylla* leaf extract (50 and 75 mg/L, respectively) in combination with GNS 60.58 mg/L, showing substantial intestinal recovery and restoration of normal tissue architecture. (K–O) Kidney histopathology: (K) Control group (C) showing normal kidney architecture; (L, M) Groups G (GNS 60.58 mg/L) and G1 (*T. aphylla* 25 mg/L combined with GNS 60.58 mg/L; no recovery) exhibiting marked histopathological alterations, including glomerulopathy (G), haemocytic infiltration (HI), widen of lumen of renal tubule (WL), inflamed nephric tubule (I), melanomacrophage centre (MMC), vacoulation (V), degeneration of outer epithelial layer of renal tubule (DO), necrosis (N), caste formation (C), diluted bowmen space (BS); (N, O) Groups G2 and G3 treated with *T. aphylla* leaf extract (50 and 75 mg/L, respectively) in combination with GNS 60.58 mg/L, showing substantial kidney recovery and restoration of normal tissue architecture.

The histological abnormalities were reported with significantly higher scores in the groups treated with GNS. Liver was reported with inflammation, melanomacrophage centres, necrosis, dilation of sinusoids, hydropic degeneration, hepatocellular vacuolation, central vein injury, pyknotic nuclei, and karyomegaly of hepatocytes (Figure 4A–E). Kidneys were reported with glomerulopathy, necrosis, cast formation, haemocytic infiltration, presence of melanomacrophage centres, degeneration of the epithelial layer, inflammation in nephric tubules, dilation of Bowman's space, alterations in tubular lumen, vacuolations and intestine was reported with collapsed epithelial cells, destroyed intestinal integrity, degenerated goblet cells, vacuolation, intact lamina propria and necrosis (Figure 4K–O). Intestine was observed with necrosis, collapsed epithelial layer, degenerated goblet cells, intact lamina propria, vacuolation, and destroyed intestinal integrity (Figure 4F–J).

The plant *T. aphylla* treatment against GNS at medium and high doses works in ameliorating these reported abnormalities and restores the original histo‐architecture of the studied organs (Table 5).

**TABLE 5:** Histology scoring in different organs of *C. mrigala* administered with co‐treatments of GNS and extract of *T. aphylla* leaf.

Parameters/organs	Groups				
**Liver**	**C**	**G**	**G1**	**G2**	**G3**
Liver inflammation	—	+	+	—	—
Melanomacrophage centres	—	+	+	—	—
Necrosis of hepatocytes	—	+	+	—	—
Dilation of sinusoids	—	+	+	—	—
Hydropic degeneration	—	+	—	—	—
Hepatocellular vacuolation	—	+	+	—	—
Central vein injury	—	+	—	—	—
Pyknotic or karyomegaly of hepatocytes	—	+	—	—	—
**Intestine**					
Collapsed epithelial cells and	—	—	+	—	—
Destroyed intestinal integrity	—	+	—	—	—
Degenerated goblet cells	—	+	+	—	—
Vacuolation	—	+	+	—	—
Intact lamina propria	—	—	+	—	—
Necrosis	—	+	+	—	—
**Kidney**					
Glomerulopathy	—	+	+	—	—
Necrosis	—	+	+	—	—
Cast formation	—	+	+	—	—
Haemocytic infiltration	—	+	+	—	—
Melanomacrophage centre	—	—	+	—	—
Degeneration of the epithelial layer	—	+	—	—	—
Inflamed nephric tubule	—	+	+	—	—
Diluted Bowmen's space	—	+	+	—	—
Changes in the tubular lumen	—	+	—	—	—
Vacuolation	—	+	—	—	—

## 4 Discussion

*T. aphylla* has pharmacological efficacy as it has a wide range of therapeutic properties combating various health issues caused by burns, injuries, or toxic stimuli (Gul et al. 2022). The bioactive compounds found in this plant could potentially counteract the harmful effects of GNS. These compounds exhibit anti‐inflammatory and liver‐protective properties, which may help reduce the toxic impact of sodium arsenate in rats (Qadir et al. 2014; Bahramsoltani et al. 2020). Additionally, this plant has demonstrated therapeutic benefits for gastrointestinal problems (Ullah et al. 2017). Furthermore, the synthesis of silver nanoparticles from *T. aphylla* is an ecofriendly approach as an alternative to conventional methods involving toxic chemicals (Ullah et al. 2017). In this study, HPLC analysis of plant extract has been shown with a wide range of phytochemicals such as ferulic acid (Bano et al. 2023), gallic acid (Nawwar et al. 2009), vanillic acid (Mahfoudhi et al. 2014), quercetin (Nawwar et al. 2009), caffeic acid (Nawwar et al. 2009), chlorogenic acid (Paul et al. 2022), cinnamic acid (Bahramsoltani et al. 2020), p‐coumaric acid, sinapic acid (Qadir et al. 2014), malic acid (Tabet et al. 2018), oxalic acid (Khalid et al. 2017), succinic acid (Orabi et al. 2022), fumaric acid, citric acid, methyl malonic acid, malonic acid (Fatima et al. 2025; Mahfoudhi et al. 2016), and kaempferol (Ismaeel et al. 2020). These bioactive compounds may contribute to mitigating the toxicity induced by GNS in fish (*C. mrigala*). This study reported different histological abnormalities, such as liver inflammation, melanomacrophage centres, necrosis, dilation of sinusoids, hydropic degeneration, hepatocellular vacuolation, central vein injury, pyknotic nuclei, and karyomegaly of hepatocytes. GNS have been identified as hepatotoxic in several studies, which found alterations in liver tissues of mice reported by El‐Yamany et al. (2017) and Fayed et al. (2023), suggesting potential health risks associated with their use. Another study reported that intravenous injection of graphene resulted in severe pathological changes in the liver, indicating potential toxicity (Mohamed et al. 2017). The toxicity of GNS is also associated with the inflammatory response in the liver tissues of experimental animals (Sasidharan et al. 2015; Amrollahi‐Sharifabadi et al. 2018). The study of Wu et al. (2019) shows that disruption in liver zonation suggests abnormalities in liver function and metabolism. These reported observations are in line with the present study, suggesting that GNS is hepatotoxic to fish.

Studies also showed that graphene oxide can affect the integrity of the intestinal barrier, leading to an increase in permeability, disrupting intestinal functions (Nirmal et al. 2021). Graphene exposure revealed alterations in the ultrastructure of the colon, suggesting toxicity at the cellular level (Domenech et al. 2020). In this study, similar alterations were observed in the histological architecture, such as collapsed epithelial cells, destroyed intestinal integrity, degenerated goblet cells, vacuolation, intact lamina propria, and necrosis. These changes have confirmed GNS as a toxic material, aligning with prior research findings. Kidney has been reported with histological abnormalities due to GNS in experimental animals, as reported by many previous studies. A previous study reported the dose‐dependent damage in kidney tubules and glomeruli (Sasidharan et al. 2015; Amrollahi‐Sharifabadi et al. 2018). Graphene exposure damages kidney tissues, resulting in dysfunction (Sasidharan et al. 2015; Amrollahi‐Sharifabadi et al. 2018; Fernandes et al. 2018; Wu et al. 2019; Nirmal et al. 2021; Domenech et al. 2020). Prominent abnormalities were also noted in the kidney tissues, such as glomerulopathy, necrosis, cast formation, haemocytic infiltration, presence of melanomacrophage centres, degeneration of the epithelial layer, inflammation in nephric tubules, dilation of Bowman's space, alterations in tubular lumen, and vacuolation, consistent with findings from previous studies. Although the studies related to the direct effects of *T. aphylla* on the intestine are limited, *T. aphylla* is protective for the intestine, as reported in some previous studies, due to its anti‐inflammatory activities. A prior study has shown that *T. aphylla* can modulate intestinal function; its methanolic extract has been reported to act as a laxative that enhances intestinal motility in experimental animals (Patil et al. 2020). The precise mechanism underlying its therapeutic effects remains unclear; however, its antioxidative and anti‐inflammatory properties may play a role. Consistent with previous findings, our study also observed a restoration of damaged intestinal structures following the application of the ameliorative agent *T. aphylla*.

*T. aphylla* is recognised for its hepatoprotective effects, enhancing liver recovery across various experimental models. The flavonoids in the plant have been demonstrated to alleviate liver injury in mice by suppressing apoptosis, oxidative stress, and inflammation (Khan et al. 2021). Additionally, the polyphenols in *T. aphylla* have been shown to reduce hepatotoxicity induced by sodium arsenate in rats (Qadir et al. 2014). These therapeutic effects are likely due to the plant's antioxidants, which scavenge free radicals and modulate inflammatory pathways (Khan et al. 2021). Our study corroborates these findings, indicating that *T. aphylla* significantly ameliorates hepatic histological abnormalities, aligning with previous research.

Moreover, *T. aphylla* is purported to possess reno‐protective properties, although research focusing specifically on kidney health remains scarce. The broad‐spectrum benefits of this plant suggest its potential in addressing renal issues (Gul et al. 2022). Antioxidants present in the plant may improve kidney function and counteract oxidative damage by scavenging free radicals (El‐Aarag et al. 2019). Furthermore, its anti‐inflammatory properties are believed to help prevent tissue damage in the kidneys (Almatroodi et al. 2022). The present results support these statements, strengthening the plant's role in renal protection and echoing the results of prior investigations.

## 5 Conclusion and Future Prospects

In the realm of technological advancements, nanomaterials, particularly GNS, have become ubiquitous across numerous industries. While these materials offer significant benefits, their introduction into aquatic environments poses potential risks to aquatic life. Our research has focused on the mitigative capabilities of *T. aphylla* leaf extract against the toxic effects of GNS on *C. mrigala*, a species integral to aquatic ecosystems. The findings indicate that co‐treatment with this plant extract, rich in compounds like citric acid, oxalic acid, and malic acid, effectively alleviates the histopathological damages induced by GNS exposure. These results not only highlight the protective effects of *T. aphylla* against GNS toxicity but also underscore the potential of using plant‐derived substances to counteract environmental pollutants, thereby offering a sustainable approach to preserving aquatic health in the face of increasing nanomaterial proliferation.

Looking forward, further studies are essential to deepen our understanding of the protective mechanisms offered by *T. aphylla* and other phytochemical‐rich plants against various nanomaterials. It is imperative to explore the scalability of using such plant extracts in real‐world environmental settings, examining their efficacy in larger aquatic systems and under different environmental stress conditions. Additionally, research should expand to investigate the interactive effects of multiple plant extracts and the potential synergistic benefits they may provide. This could lead to the development of comprehensive strategies for managing nanomaterial pollution in aquatic environments. Finally, regulatory frameworks need to be informed by such research to ensure that the integration of nanotechnologies into industrial applications is balanced with environmental safety and sustainability.

## Author Contributions

**Muhammad Asad**: conceptualisation, investigation, formal analysis, writing – original draft; **Kashif Ali**: formal analysis, data curation, writing – original draft; **Tehseen Fatima**: investigation, data curation, resources; **Nagina Rehman**: methodology, visualisation; **Abeer Kazmi**: writing – review & editing; **Md. Fakhrul Islam**: writing – review & editing; **Aneela Nijabat**: investigation, methodology; **Asma Ashraf**: data curation, formal analysis, visualisation; **Ihsan Ullah**: methodology, data curation; **Ayesha Siddiqa**: methodology, visualisation; **Juan Pedro Luna‐Arias**: writing – review & editing; **Gabriela Medina‐Pérez**: writing – review & editing. **Amir Ali**: writing – review & editing. All authors have read and approved the final version of the manuscript.

## Funding

The authors have nothing to report.

## Ethics Statement

The Ethics Committee on Animal Experimentation in the Department of Zoology, University of Education Lahore (Jauharabad campus), has approved the experiment conducted on the fish species *Cirrhinus mrigala*. The approval, documented as Approval No UE/JBD/ZOOL/2023/16, was issued on 2 February, 2023.

## Conflicts of Interest

Authors declare no conflicts of interest.

## Data Availability

The data that support the findings of this study are available from the corresponding author upon reasonable request.
